# Supplementary figures and images for: Molecular detection and characterisation of the first Japanese encephalitis virus belonging to genotype IV acquired in Australia
Source: PLoS Negl Trop Dis. 2022 Nov 21;16(11):e0010754. doi: 10.1371/journal.pntd.0010754 (PMC9721490; doi:10.1371/journal.pntd.0010754)

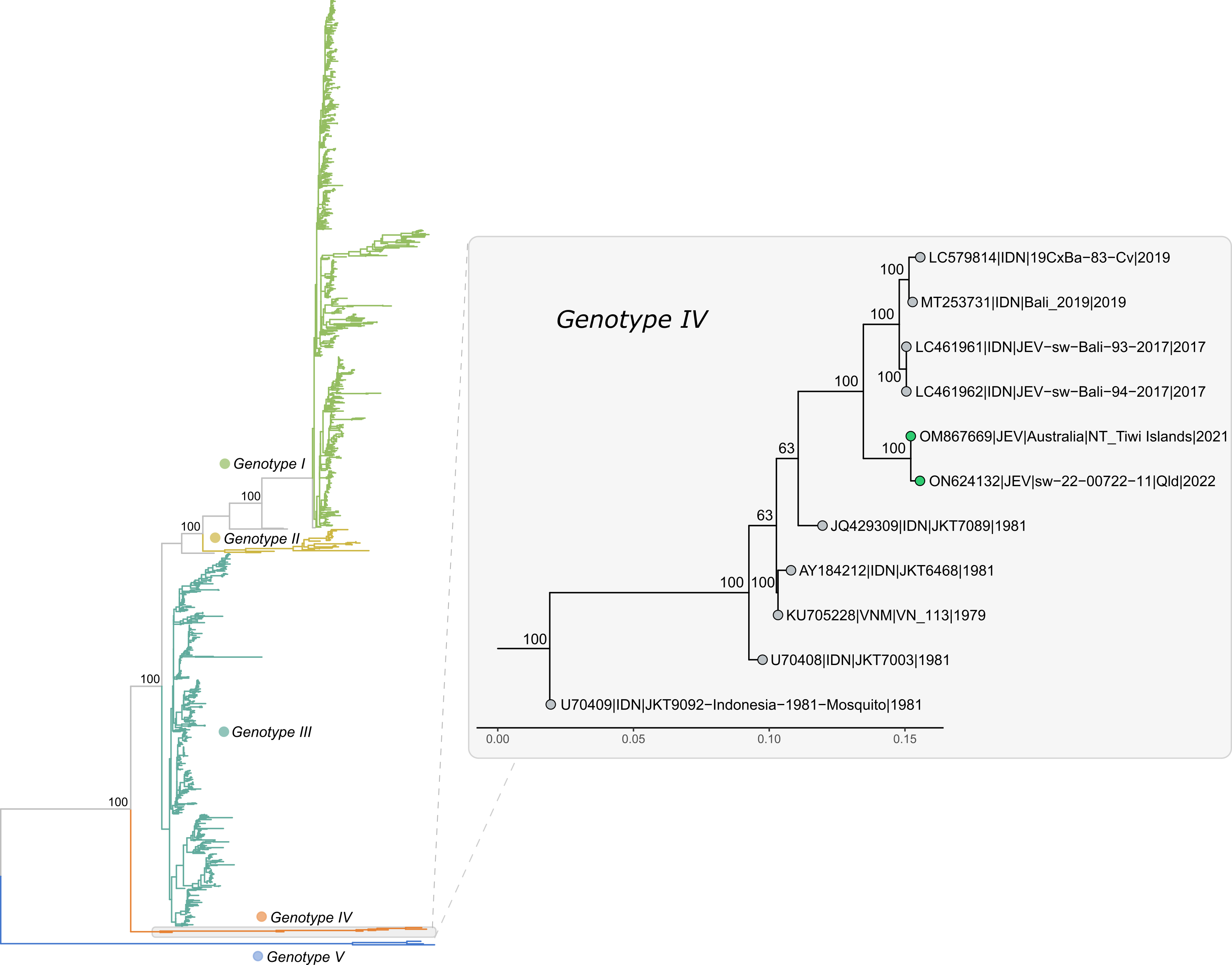

Supplement: S1 Fig — The TIM2 model with gamma rate heterogeneity was chosen as the most appropriate model by IQ-TREE v.2.0.6. The results from 1000 bootstrap replicates are given on the nodes and the scale represents the number of nucleotide substitutions per site. (TIF) [file pntd.0010754.s001.tif]

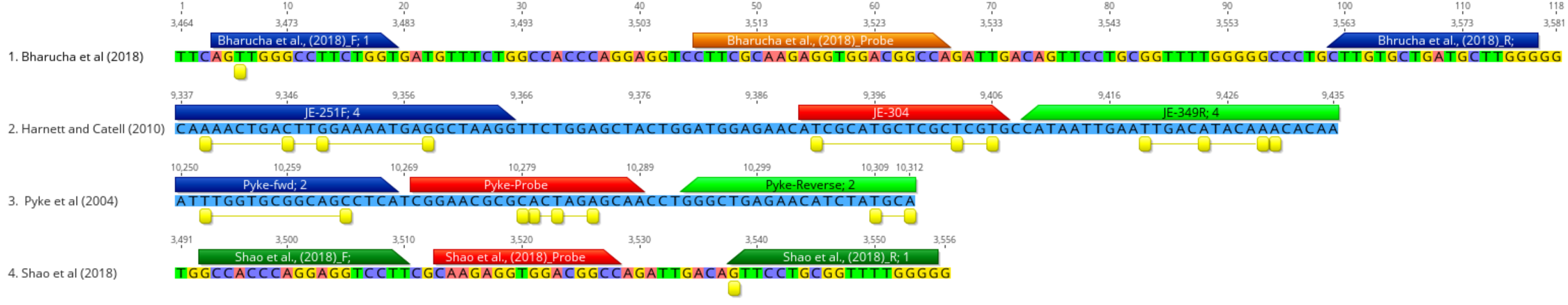

Supplement: S2 Fig — Primer and probe positions are shown according to the sequence of the JEV/Australia/NT_Tiwi Islands/2021 genome (OM867669). JEV RT-PCR assays used at PWLM comprised primers and probes in alignments 1 and 2 [34,35]. JEV RT-PCR assays used at ACDP comprised primers and probes in alignments 3 and 4 [32,33]. (TIF) [file pntd.0010754.s002.tif]
