# Supplementary material for: Molecular detection and characterisation of the first Japanese encephalitis virus belonging to genotype IV acquired in Australia
Source: PLoS Negl Trop Dis. 2022 Nov 21;16(11):e0010754. doi: 10.1371/journal.pntd.0010754 (PMC9721490; doi:10.1371/journal.pntd.0010754)
Supplement: S1 Table — (DOCX) [file pntd.0010754.s003.docx]

| Year | Location^a^ | Mosquito species | Genotype (G) | Reference | Clinical disease |
| --- | --- | --- | --- | --- | --- |
| 1995 | Badu Island, QLD | *Culex annulirostris* | GII | [1] | Three encephalitis cases, two fatal |
| 1998 | Badu Island, QLD | *Cx. annulirostris, Ae. vigilax* | GII | [2] | One encephalitis case |
|  | Mitchell River mouth, Cape York, QLD | No JEV detected | GII | [2] | One encephalitis case |
| 2000 | Badu Island, QLD | *Cx. gelidus* | GI | [3] | None reported |
| 2004 | Badu Island, QLD | Isolated from sentinel pig | GI | [4] | None reported |
|  | Northern Peninsular Area, Cape York, QLD | *Cx. annulirostris, Cx.*  *palpalis* | GI | [4] | None reported |
| 2021 | Tiwi Islands, NT,  VIC^b^ | No JEV detected | GIV | [5, 6] | 2 cases, 1 fatal |
| 2022 | NSW, QLD, SA, VIC, NT | *Cx. annulirostris* | GIV | [6, 7] | 40 cases, 6 fatal |

**S1 Table:** Japanese encephalitis virus detections in Australia.

^a^QLD, Queensland; NT, Northern Territory; NSW, New South Wales; SA, South Australia; VIC, Victoria.

^b^The case attributed to VIC in 2021 was acquired in the NT from a traveller from VIC and was identified retrospectively (Prof. B. Currie, Royal Darwin Hospital, unpublished).

**References**

1. Hanna JN, Ritchie SA, Phillips DA, Shield J, Bailey MC, Mackenzie JS, et al. An outbreak of Japanese encephalitis in the Torres Strait, Australia, 1995. Med J Aust. 1996;165(5):256-60.
2. Hanna JN, Ritchie SA, Phillips DA, Lee JM, Hills SL, van den Hurk AF, et al. Japanese encephalitis in north Queensland, Australia, 1998. Med J Aust. 1999;170(11):533-6. PubMed PMID: 10397044.
3. Pyke AT, Williams DT, Nisbet DJ, van den Hurk AF, Taylor CT, Johansen CA, et al. The appearance of a second genotype of Japanese encephalitis virus in the Australasian region. Am J Trop Med Hyg. 2001;65(6):747-53. PubMed PMID: 11791969.
4. Van Den Hurk AF, Montgomery BL, Northill JA, Smith IL, Zborowski P, Ritchie SA, et al. Short report: the first isolation of Japanese encephalitis virus from mosquitoes collected from mainland Australia. Am J Trop Med Hyg. 2006;75(1):21-5. PubMed PMID: 16837702.
5. Waller C, Tiemensma, M, Krause, VL, Williams, DT, Baird, RW, Currie, BJ. Japanese encephalitis outbreak, Australia; the sentinel case. New Eng J Med. 2022;387(7):661-2. doi: 10.1056/NEJMc2207004.
6. Australian Government Department of Health and Aged Care. Health alerts: Japanese encephalitis virus 2022 [cited 8^th^ November 2022. Available from: https://www.health.gov.au/health-alerts/japanese-encephalitis-virus-jev/about#current-status.
7. Furuya-Kanamori L, Gyawali N, Mills DJ, Hugo LE, Devine GJ, Lau CL. The Emergence of Japanese Encephalitis in Australia and the Implications for a Vaccination Strategy. Trop Med Infect Dis. 2022;7(6). Epub 2022/06/24. doi: 10.3390/tropicalmed7060085. PubMed PMID: 35736964.
