# Supplementary material for: Molecular detection and characterisation of the first Japanese encephalitis virus belonging to genotype IV acquired in Australia
Source: PLoS Negl Trop Dis. 2022 Nov 21;16(11):e0010754. doi: 10.1371/journal.pntd.0010754 (PMC9721490; doi:10.1371/journal.pntd.0010754)
Supplement: S2 Table — (DOCX) [file pntd.0010754.s004.docx]

**S2 Table.** Primer scheme used for the amplification of JEV 1600bp amplicon sequencing.

| **Name** | **Minimum*** | **Maximum*** | **Direction** | **Sequence (5’ to 3’)** | **Length** |
| --- | --- | --- | --- | --- | --- |
| JEV_1_F | 3 | 28 | forward | AGTTTATCTGTGTGAACTTCTTGRTT | 26 |
| JEV_1_R | 1576 | 1597 | reverse | ACTTTGAGCCCACAGTCATGAC | 22 |
| JEV_2_F | 1493 | 1514 | forward | CAATAACCCTGAAGCTTGGCGA | 22 |
| JEV_2_R | 3064 | 3085 | reverse | GGTGACTCTCGATCCAGTACGA | 22 |
| JEV_3_F | 2996 | 3017 | forward | CTGATGAGTGCGATGGAACGAT | 22 |
| JEV_3_R | 4562 | 4583 | reverse | AGAGTGAGCCAGTAACCAAACG | 22 |
| JEV_4_F | 4493 | 4514 | forward | AGGTTTGGGTCTTACGCATGTC | 22 |
| JEV_4_R | 6060 | 6081 | reverse | GATTTTTGCTTCCGTCCAGTGG | 22 |
| JEV_5_F | 5490 | 5511 | forward | GCGGGGTTACATTGCAACAAAA | 22 |
| JEV_5_R | 7057 | 7078 | reverse | TGTGTTTCAAGAGAGGCGTCAG | 22 |
| JEV_6_F | 6866 | 6887 | forward | TCATCTGTGTCCTGACTGTGGT | 22 |
| JEV_6_F | 8332 | 8353 | reverse | CATTACCAGCTGCTCCGCTAAC | 22 |
| JEV_7_F | 7790 | 7809 | forward | GTAGAGCCAGGCGTGAGAAC | 20 |
| JEV_7_R | 9561 | 9580 | reverse | CCACATGTTGCGGTCCAATG | 20 |
| JEV_8_F | 9363 | 9382 | forward | GCTCGCTCGTGCCATAATTG | 20 |
| JEV_8_F.2 | 9440 | 9459 | forward | CMGTGATGGACGTGATATCA | 20 |
| JEV_8_R | 10924 | 10943 | reverse | ACCACCAGCCACATACTTCG | 20 |

*Primer positions were all set according to the reference sequence of the Australian G4 JEV strain OM867669
