# Supplementary material for: Molecular detection and characterisation of the first Japanese encephalitis virus belonging to genotype IV acquired in Australia
Source: PLoS Negl Trop Dis. 2022 Nov 21;16(11):e0010754. doi: 10.1371/journal.pntd.0010754 (PMC9721490; doi:10.1371/journal.pntd.0010754)
Supplement: S3 Table — (DOCX) [file pntd.0010754.s005.docx]

**S3 Table.** Positively selected sites within the JEV/Australia/NT_Tiwi Islands/2021 genome.

| Protein | Position | Method | P-value |
| --- | --- | --- | --- |
| PrM | 85 | MEME | 0.00 |
| E | 126 | FEL | 0.08 |
| E | 194 | FEL | 0.05 |
| NS1 | 105 | FEL | 0.08 |
| NS2a | 134 | MEME | 0.08 |
| NS3 | 436 | MEME | 0.06 |
| NS3 | 487 | MEME | 0.08 |
| NS4a | 5 | FEL | 0.05 |
| NS5 | 72 | FEL | 0.07 |
| NS5 | 284 | FEL | 0.04 |
| NS5 | 390 | FEL | 0.09 |
| NS5 | 643 | FEL | 0.01 |
