# Supplementary material for: Molecular detection and characterisation of the first Japanese encephalitis virus belonging to genotype IV acquired in Australia
Source: PLoS Negl Trop Dis. 2022 Nov 21;16(11):e0010754. doi: 10.1371/journal.pntd.0010754 (PMC9721490; doi:10.1371/journal.pntd.0010754)
Supplement: Attachment — Submitted filename: Characterisation of Australian Japanese encephalitis virus genotype IV.docx [file pntd.0010754.s006.docx]

Sikazwe et al. attempted to isolate the virus from clinical specimens, and successfully extracted by the genome-wide nucleic acid of the virus. Subsequently, they made clear that the separation of the virus is GIV JEV through whole genome sequencing and phylogenetic tree analysis. The GIV JEV strains first time emerged in Australia, and this is the second time confirmed fatal human infection GIV JEV strains. Study findings have significant public health implications, but there are some imperfections Although researchers have tried several methods to isolate JEV, they did not continue to isolate the virus. In addition, new efforts should be directed in an attempt to isolate JEV using other virus isolation methods. To better control JE and obtain more study materials of JEV, obtaining more information of viral characteristics (such as transmissibility and pathogenicity) is necessary. To obtain more accurate and reproducible results various virus identification methods should be used, such as RT-PCR and indirect immunofluorescence assay (IFA). Viral strains should be isolated from clinical samples as soon as possible and use immunological approaches to prove the isolates are GIV JE virus will qualify this paper for publication in the Frontiers PLoS Neglected Tropical Diseases.

Q1: Having failed to isolate the virus, why not try other methods? It is recommended to use human cells susceptible to JEV or inoculate suckling mice to isolate viruses.

Line 29 change "Real-time RT-qPCR" to" Reverse transcription quantitative PCR (RT-qPCR)"

Line 143 What is the antibiotic concentration used in this test?

Line 151 recommend changing “real-time reverse transcriptase PCR (RT-PCR)” to “Reverse transcription quantitative PCR (RT-qPCR)”, The abbreviation RT-PCR is easily confused with the abbreviation reverse transcription PCR, it is suggested that the abbreviation of RT-qPCR should be used uniformly throughout the text.

Line 217 The incubation time can be extended, and more virus was allowed to adsorb onto the cells. (Such as 2h)

Line 228 what dose did the embryonated chicken eggs receive?

Minor points:

Line 382 change "S1 Fig" to "Fig S1"

Line 398 change " S3 Table " to "Table S3"

Line 405 and line 560 change "S1 Fig" to "Fig S1"

Line 445 and line 573 change "S3 Fig" to "Fig S3"

Line 506 and line 564 change "S2 Fig" to "Fig S2"

Line 569 change " S1 Table " to "Table S1"

Line 570 change " S2 Table " to "Table S2"
