# Supplementary material for: Molecular detection and characterisation of the first Japanese encephalitis virus belonging to genotype IV acquired in Australia
Source: PLoS Negl Trop Dis. 2022 Nov 21;16(11):e0010754. doi: 10.1371/journal.pntd.0010754 (PMC9721490; doi:10.1371/journal.pntd.0010754)
Supplement: Attachment — Submitted filename: PNTD-D-22-01068_Author response.docx [file pntd.0010754.s007.docx]

**Author responses for manuscript submission PNTD-D-22-01068: "Molecular detection and characterisation of the first Japanese encephalitis virus belonging to genotype IV in Australia"**

**Reviewer #1:**

No comments provided.

**Reviewer #2:**

This paper looks ready to be published and contains interesting data.

*Author Response:* We thank the reviewer for their encouraging comments.

**Reviewer #3:**

In the manuscript “Molecular detection and and characterisation of the first Japanese encephalitis virus belonging to genotype IV in Australia” (PNTD-D-22-01068), Sikazwe et al. reports the detection and whole genome sequencing of Japanese encephalitis virus (JEV) RNA recovered from a fatal case of JE that occurred in a resident of the Tiwi Islands, in the Northern Territory of Australia in 2021. The authors then perform an evolutionary analysis to examine the link between this new genotype IV sequence (first detection of genotype IV of JEV in Australia), other genotype IV sequences from Vietnam and Indonesia detected from 1979─2019, and a genotype IV sequence recovered from a large JE outbreak in southeastern Australia in 2022. The close phylogenetic relationship between the Tiwi Island sequence and recent Indonesian sequences suggest that Indonesia might be the origin of genotype IV viruses currently circulating in Australia.

This manuscript is very well-written, and the study was well-executed and scientifically sound. The introduction includes all relevant information necessary to understand the study, the methods are comprehensive, the results are well-organized, and the discussion nicely discusses possible origins of genotype IV sequences currently circulating in Australia, and the need for JEV vector competence studies and ongoing surveillance for the virus. Listed below are a few very minor comments.

Specific comments

Lines 132-136 and 228-233: Prior to reviewing this manuscript, I was not familiar with the utility of using embryonated chicken eggs to isolate avian-associated flaviviruses. Please consider adding some references describing this procedure and its relative success compared to other methods of isolation (i.e., cell culture and intracranial inoculation of suckling mice).

*Author Response:*

*We have modified the Diagnostic Virology section of the results, including the additional text to address this comment:*

*‘Virus isolation attempts using ECEs, mammalian cells (Vero) and mosquito cells (C6/36) followed by passage in mammalian cell lines (Vero, BHK-21) were unsuccessful. We also attempted isolation from the right thalamus specimen via inoculation of the CAMs of ECEs, a culture system that has previously been shown to be more sensitive than cell culture and suckling mouse inoculation methods for isolating MVEV, a closely related flavivirus to JEV, from human brain specimens [47, 48]. Unfortunately, virus was also unable to be isolated using this ECE culture system.’*

Lines 346-349 and Table 1: It is interesting that the Shao et al., 2018 RT-PCR assay detected JEV RNA in the right thalmus tissue sample but in no other tissue sample types. Do you happen to have an explanation for this finding (e.g., sequence mismatch in the primer-binding site)?

*Author Response: Only the right thalamus tissue sample was available to test using the Shao et al. assay. This is indicated in the table for other tissue samples by ‘NT, not tested’. An additional sentence to clarify this has been added to the Diagnostic Virology section of the Results.*

Lines 418-420: Has regular surveillance for JEV in mosquitoes, birds, and/or pigs been conducted in Timor-Leste, West Papua, and Papua New Guinea? After reading this sentence, I am unclear if regular surveillance for JEV is conducted, and the virus has not been detected or if regular surveillance has not been conducted. Please clarify.

*Author Response: No regular surveillance for JEV has been undertaken in Papua New Guinea, West Papua or Timor Leste. Most information has been obtained through sporadic and opportunistic sampling in PNG and West Papua as part of research programs. No studies have been reported from Timor Leste that we are aware of. We have attempted to clarify this section further, as follows:*

*‘Regional surveillance for JEV will also be pivotal in understanding the origins of the Tiwi Islands strain, and the extent of genetic diversity and geographic spread of JEV GIV. To date, our knowledge of JEV activity in the countries neighbouring Australia’s northern borders has been informed by case reports or through sporadic or opportunistic sampling and testing as part of surveillance programs or discrete research studies [17-19, 21]. Thus, there are no published reports of the detection of JEV GIV from Timor-Leste, West Papua, or Papua New Guinea, all of which lie to the north of Australia.’*

Figures 2 and 3, and Supplementary figure 1: Please include bootstrap support values for the genotype-defining nodes.

*Author Response: Bootstrap values have been added to the revised figures (Fig 2 and S1 Fig). The MCC tree shown in Fig 3 indicates posterior probability that are >0.7 at all nodes, indicated by asterisks. For clarity, the authors would prefer to keep the format of this figure unchanged. In the process of reviewing Fig 3, it was noted that asterisks had been omitted for the basal and GI/GII nodes. Fig 3 has been amended to add these asterisks.*

*Note that a minor change to the Fig 3 caption has als obeen made to clarify the position of the asterisks: ‘Posterior probability values of >0.70 are presented adjacent to nodes as indicated by asterisks.’*

**Reviewer #4 (comments attached to decision email sent 26/09/22)**

Sikazwe et al. attempted to isolate the virus from clinical specimens, and successfully extracted by the genome-wide nucleic acid of the virus. Subsequently, they made clear that the separation of the virus is GIV JEV through whole genome sequencing and phylogenetic tree analysis. The GIV JEV strains first time emerged in Australia, and this is the second time confirmed fatal human infection GIV JEV strains. Study findings have significant public health implications, but there are some imperfections Although researchers have tried several methods to isolate JEV, they did not continue to isolate the virus. In addition, new efforts should be directed in an attempt to isolate JEV using other virus isolation methods. To better control JE and obtain more study materials of JEV, obtaining more information of viral characteristics (such as transmissibility and pathogenicity) is necessary. To obtain more accurate and reproducible results various virus identification methods should be used, such as RT-PCR and indirect immunofluorescence assay (IFA). Viral strains should be isolated from clinical samples as soon as possible and use immunological approaches to prove the isolates are GIV JE virus will qualify this paper for publication in the Frontiers PLoS Neglected Tropical Diseases.

Q1: Having failed to isolate the virus, why not try other methods? It is recommended to use human cells susceptible to JEV or inoculate suckling mice to isolate viruses.

*Author Response: We followed a standard method for isolating arboviruses in cell culture using the Aedes C6/36 clone and mammalian cell lines. This approach has been employed successfully for isolating flaviviruses from vectors and mammalian hosts, including humans (eg Johansen, CA, et al., Am. J. Trop. Med. Hyg., 62, 631, 2000; Pyke AT, et al., Trop Med Infect Dis. 2020, 5(3):133; also reviewed in Pham D, et al. Pathology. 2022, 54(6):669-677 and Morita K, et al. Rev. Sci. Tech. Off. Int. Epiz., 2015, 34 (2), 441-452). We also acknowledge that human cells can also be employed for JEV isolation from clinical specimens; however, at the time of testing, these were not validated for JEV isolation in our laboratories. Unfortunately, neither laboratory involved held animal ethics approvals for performing the SMIC method at the time of testing; however, this is acknowledged as a sensitive culture system. Due to the limited amount of clinical specimen available, we prioritised remaining samples for molecular characterisation.*

Line 29 change "Real-time RT-qPCR" to" Reverse transcription quantitative PCR (RT-qPCR)"

*Author Response: This change has been made.*

Line 143 What is the antibiotic concentration used in this test?

*Author Response: The final concentration was* *400 IU/ml of penicillin and 400 µg/ml of streptomycin. This information has been added to this section of the methods.*

Line 151 recommend changing “real-time reverse transcriptase PCR (RT-PCR)” to “Reverse transcription quantitative PCR (RT-qPCR)”, The abbreviation RT-PCR is easily confused with the abbreviation reverse transcription PCR, it is suggested that the abbreviation of RT-qPCR should be used uniformly throughout the text.

*Author Response: This change has been made, as recommended.*

Line 217 The incubation time can be extended, and more virus was allowed to adsorb onto the cells. (Such as 2h)

*Author Response: We agree with the reviewer that extending the incubation time can result in greater levels of virus adsorption. However, due to limited specimen available for virus isolation, repeat testing was not performed. Given the negative results using different cell lines and culture systems in two different laboratories, the authors concluded that cultivable levels of viable virus were not present in the specimens tested. The remaining specimens were prioritised for genome sequencing.*

Line 228 what dose did the embryonated chicken eggs receive?

*Author Response: ECEs were inoculated with 200ul of sample, as indicated in the methods section.*

Minor points:

Line 382 change "S1 Fig" to "Fig S1"

Line 398 change " S3 Table " to "Table S3"

Line 405 and line 560 change "S1 Fig" to "Fig S1"

Line 445 and line 573 change "S3 Fig" to "Fig S3"

Line 506 and line 564 change "S2 Fig" to "Fig S2"

Line 569 change " S1 Table " to "Table S1"

Line 570 change " S2 Table " to "Table S2"

*Author Response to Minor points: The PLoS NTD guidelines indicate the formatting that we have used for referring to supplementary material. A check of formatting used in the current issue confirmed this.*

*Author note: Additional amendments have been made as follows:*

i. *To reference 26. It was noticed that this citation was incomplete and this has now been corrected.*
ii. *In S1 Table, it was noted that the incorrect citation was made for the year 2004. This has been corrected to reference [53; van den Hurk et al., 2006].*
iii. *Fig 1 caption. To address editor comments and request to clarify the licence used to generate the map, the following statements have been added: ‘The map was created in QGIS version 3.26.2-Buenos Aires (QGIS Development Team (2022). QGIS Geographic Information System. Open Source Geospatial Foundation Project. http://qgis.osgeo.org). The base layer of the map used to generate this figure was downloaded from https://www.abs.gov.au/statistics/standards/australian-statistical-geography-standard-asgs-edition-3/jul2021-jun2026/access-and-downloads/digital-boundary-files and is licensed under a Creative Commons Attribution 4.0 International licence.’*
